# Amikacin exposure in MDR-TB patients in Uganda: Revisiting old drugs in a new era of resistance – A pharmacokinetic assessment

**DOI:** 10.1016/j.nmni.2026.101734

**Published:** 2026-02-27

**Authors:** Jan Hongler, Sabine Haller, Akello Susan Adakun, Nina Lutz, Allan Buzibye, Marisa Kälin, Barbara Castelnuovo, Christine Sekaggya-Wiltshire, George Abongomera, Alexander Jetter, Jan Fehr

**Affiliations:** a Department of Public and Global Health, Epidemiology, Biostatistics and Prevention Institute, University of Zurich, Zurich, Switzerland; b Infectious Diseases Institute, College of Health Sciences, Makerere University, Uganda; c Division of Infectious Diseases, Infection Prevention and Travel Medicine, HOCH Cantonal Hospital St. Gallen, St. Gallen, Switzerland; d National Tuberculosis Treatment Centre, Mulago National Referral and Teaching Hospital, Kampala, Uganda; e Department of Infectious Diseases and Hospital Epidemiology, University Hospital Zurich, Zurich, Switzerland; f Swiss Centre for International Health, Swiss Tropical and Public Health Institute, Basel, Switzerland; g Tox Info Suisse, Swiss National Poison Centre, Associated Institute of the University of Zurich, and Department of Clinical Pharmacology and Toxicology, University Hospital Zurich and University of Zurich, Zurich, Switzerland

**Keywords:** MDR**-**Tuberculosis, Aminoglycoside, Pharmacokinetics, Toxicity, Uganda

## Abstract

**Background:**

Amid rising resistance to bedaquiline, aminoglycosides remain an important fallback option for multidrug-resistant tuberculosis (MDR-TB) treatment, particularly in high-burden settings. Their use is limited by nephro- and ototoxicity, which is associated with cumulative drug exposure. In this study we investigated amikacin exposure in Ugandan MDR-TB patients using a validated limited sampling strategy and compared the results to previously published data from a Western European cohort.

**Methods:**

In this single-centre prospective observational study, 29 MDR-TB patients received amikacin at a dose of 10-15 mg/kg. Serum levels were measured on day 30 at 1, 4 and 5 h post-administration using liquid chromatography/mass spectrometry. Individual concentration-time curves were modelled using a one-compartment model and compared to a Dutch population-pharmacokinetic (PK) model.

**Results:**

Twenty patients had complete PK data. Patients received a median amikacin dose of 10.9 (IQR 10 - 14.9) mg/kg; clearance was 4.79 L/h (IQR 4.03 - 5.75), volume of distribution (Vd) 16.3 L (IQR 14.07 – 21.49), AUC_0-24h_ 125.15 h x mg/l (IQR 106.73 – 174.46), maximum serum concentration (Cmax) 27.8 mg/l (IQR 22.9 – 48.7).

**Conclusions:**

This population-PK study shows that major differences in PK between Ugandan MDR-TB patients and those in the Global North are unlikely. Our findings reinforce the suitability of a one-compartment model for therapeutic drug monitoring in both high- and low-resource settings. Readily obtained aminoglycoside PK parameters in a limited resource setting facilitate future efforts in optimizing drug exposure with minimal toxicities, in the population most affected by the pandemic of TB.

## 1 Background

The tuberculosis (TB) pandemic constitutes an ongoing global public health emergency. In the year 2024, an estimated 10.7 million individuals developed active TB, and more than 1.2 million people succumbed to the disease, making TB again the world's leading cause of death from a single infectious agent [1].

Despite ongoing public health efforts, the emergence and spread of multidrug-resistant and rifampicin-resistant TB (MDR/RR-TB) continues to undermine global TB control strategies [1]. In 2024, approximately 390,000 new cases of MDR/RR-TB were reported globally; however, only 42% of these patients received appropriate treatment [1]. In high-burden countries such as Uganda, the prevalence of MDR/RR-TB is reported to range between 2% and 12%, underscoring the urgent need for effective and sustainable treatment options [[2], [3], [4]].

The introduction of novel anti-TB agents such as bedaquiline and pretomanid, and the widespread rollout of a novel, shorter bedaquiline–linezolid–pretomanid (BPaL)-based treatment regimen, has markedly advanced the treatment of MDR/RR- TB, offering improved outcomes and better tolerability compared to traditional aminoglycoside-based regimens [5,6]. However, growing evidence indicates that resistance to bedaquiline is on the rise. A recent meta-analysis estimated the global prevalence of bedaquiline resistance at approximately 5.7% (95% CI: 3.6–8.3%), with regional hotspots such as South Africa and Mozambique reporting prevalence rates exceeding 10% [[7], [8], [9]]. Resistance may emerge both through primary transmission and through acquired mutations under selective pressure, driven by subtherapeutic bedaquiline exposure linked to its prolonged elimination half-life [10,11]. The implications are substantial: therapeutic options become more limited, and established regimens such as BPaL have been shown to lose efficacy [12,13].

In this context, aminoglycosides, once considered a cornerstone of MDR-/RR-TB therapy, are poised to regain clinical relevance amid a lack of viable treatment alternatives. Their previous de-prioritization in standardized regimens was mainly attributed to their adverse toxicity profile [5,[14], [15], [16], [17]]. With their narrow therapeutic margin, aminoglycosides pose a significant risk of irreversible nephro- and ototoxicity. This is especially true when dosed by weight bands, the standard practice for TB-drugs. This method can, while simplifying dosing, result in substantial variability in drug exposure. Patients at the upper or lower limits of weight bands may receive doses that are either insufficient or excessively high, increasing the risk of aminoglycoside related toxicities. For amikacin, the maximum serum concentration over the minimum inhibitory concentration (Cmax/MIC) has been generally accepted to be the most important pharmacokinetic-pharmacodynamic (PK/PD) parameter for drug efficacy, reflecting the concept of concentration-dependent killing for aminoglycosides [18]. Conventional aminoglycoside dosing regimens aim to achieve specific Cmax levels while keeping trough blood concentrations (Cmin) low to reduce the risk of toxicity. Treatment with aminoglycosides leads to nephro- and ototoxicity in approximately 8 to 37% of patients, worsening with prolonged exposure and higher doses [[14], [15], [16], [17],^19^]. Contrary to clinical monitoring practice, amikacin-related toxicities seem to be neither dependent on trough, nor on peak concentrations of amikacin, but are associated with the cumulative area under the curve (AUC) in the individual patient [16,20]. However, the measurement of the AUC requires multiple samplings following drug application, which is not feasible in daily practice. Therefore, limited sampling strategies (LSS) have been developed to estimate the AUC based on fewer actual measurements [21]. One LSS derived from population PK modelling was created in a TB centre in the Netherlands to predict the median 24-h area under the concentration–time curve (AUC_0-24_) by using one, two, or three concentrations at predefined sampling time points [22]. In other publications comparable schemes have been proposed [23]. The limited sampling scheme used in this study was selected based on the robustness of available data regarding its predictive performance. To our knowledge, the use of an LSS to estimate amikacin exposure in sub-Saharan Africa, where the TB burden is highest, has not been published.

We therefore investigated amikacin pharmacokinetics in patients with MDR/RR-TB in Uganda using a limited sampling strategy. The aim was to characterize drug exposure in the population to be treated and to evaluate whether pharmacokinetic parameters and modeling approaches established predominantly in populations from the Global North are applicable and transferable to this high-burden sub-Saharan African setting.

## 2 Methods

### 2.1 Patients

This single-centre prospective observational study was conducted at the National Tuberculosis Treatment Centre, based at Mulago Hospital in Kampala, Uganda. Patients with mycobacteriologically confirmed RR- or MDR-TB (positive sputum smear for acid fast bacilli (AFB), GeneXpert MTB/RIF assay, sputum culture) were screened and consecutively enrolled between August 2019 and February 2020. Inclusion criteria were age ≥18 years and eligibility for a treatment regimen containing amikacin according to the Ugandan National Treatment Guidelines at the time of enrolment (2018). MDR/RR-TB was diagnosed using GeneXpert MTB/RIF or solid culture on Lowenstein-Jensen medium, and drug susceptibility testing on either sputum or extra-pulmonary material. Patients were excluded if they were not eligible to receive amikacin as defined by the national treatment guidelines or were enrolled in any other ongoing clinical trial.

Ethical approval was given by Mulago Hospital Research & Ethics Committee (MHREC 1520) and the Uganda National Council of Science and Technology (HS402ES). Approval was given by the National Drug Authority. Written informed consent was obtained from all participants.

### 2.2 Treatment

All subjects received treatment for MDR-TB containing amikacin during the intensive phase of treatment. Amikacin was administered intramuscularly at a dose of 10-15 mg/kg, with a maximum dose of 1000 mg per day. Treatment regimens followed the Uganda National Guidelines for Drug-Resistant Tuberculosis [24,25], which are based on the WHO guidelines with a modification in dosing, as specified in the national protocol [5].

### 2.3 Study procedures

At enrolment, demographic data, medical history, and TB treatment history was obtained, and a physical examination was conducted. TB treatment was started at the day of enrolment. Venous whole-blood samples were drawn at enrolment and at day 30. At day 30, additional blood samples for amikacin PK were taken at 1, 4 and 5 h after administration of amikacin. The samples for amikacin quantification were centrifuged, and the serum was stored at −80 °C until analysis. Serum creatinine measurements and pure tone audiometry for both ears were performed at baseline and at day 30.

Kidney function was evaluated by serum creatinine measurement and calculation of the estimated glomerular filtration rate (eGFR) using the CKD-EPI creatinine equation [26].

### 2.4 Pharmacokinetics

In-house liquid chromatography mass spectrometry (LC-MS) was used to determine the serum concentration of amikacin (Appendix 1). The PK parameters clearance (Cl), volume of distribution (Vd), AUC_0-24h_ and Cmax were calculated using a one compartment model as it is included in MW\Pharm, Mediware a.s., Praha, Czech Republic.

## 3 Results

A total of 29 patients were identified and enrolled in this study between August 2019 and February 2020. Patient characteristics are presented in Table 1. The diagnosis of RR/MDR-TB was established through positive GeneXpert MTB/Rif in 28/29 patients (96.6%) and sputum culture in 10/29 (34%). GeneXpert MTB/RIF was positive in all patients with positive culture results. One patient was diagnosed based on clinical suspicion as an MDR-TB relapse, with no results on GeneXpert, and mycobacterial culture showing contamination. TB manifestation was pulmonary in all patients.

**Table 1:** Baseline characteristics of participants who were included in the PK analysis (n = 20).

Characteristics	
Male gender (%)	13 (65)
Age in years, median (Range)	37 (22 - 55)
Nationality, Ugandan (%)	19 (95)
Weight in kg, median (Range)	51.75 (34 – 79.5)
Body Mass Index (in kg/m^2^), median (Range)	19.3 (14.4 -29.9)
Living with HIV (%)	9 (45)
On ART (% of above)	9 (100)
Diabetes mellitus	0
Arterial hypertension	0
Site of disease	
-Pulmonary (%)	20 (100)
Previous Tuberculosis (%)	
- Yes	9 (45)
Resistance by culture (%)	
-RR	2 (10)
-MDR	3 (15)
-No culture available	15 (75)
-GeneXpert available	20 (100)
Serum creatinine in mg/dL, median (Range)	0.6 (0.43 - 1.1)
eGFR in ml/min/1,73 m^2^, median (Range)	123.3 (106.3 - 146.2)
- > 90	19 (96)^a^
Amikacin dose/kg body weight in mg/kg, median (Range)	10.9 (9.4 -18.9)

Standard deviation (SD), interquartile range (Range), antiretroviral treatment (ART), estimated glomerular filtration rate (eGFR), Rifampicin resistant (RR), Multidrug-resistant (MDR).

a One patient had missing data.

**Fig. 1:**
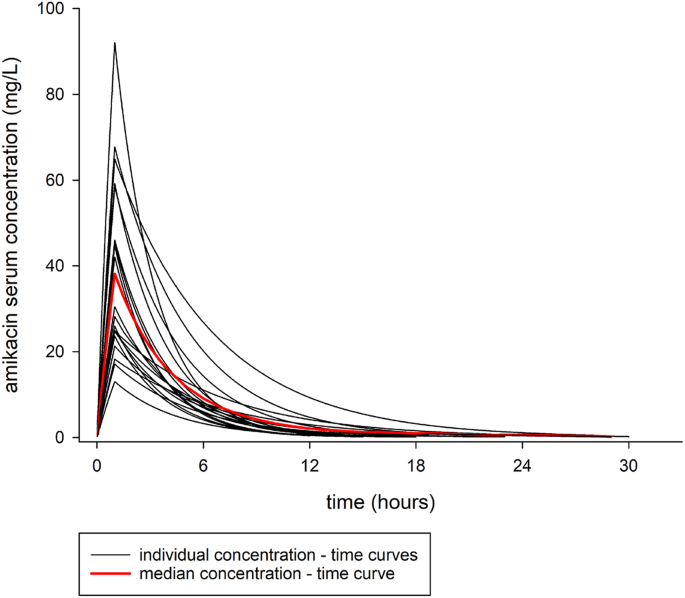
Simulated amikacin serum concentration versus time curves for all patients with complete PK information. Red line: median concentration.

One patient withdrew from treatment; another patient was excluded because he developed a second-degree hearing loss and was therefore switched to an aminoglycoside free treatment regimen before sampling on day 30. Seven patients were excluded because whole blood samples for PK analysis and results were either missing, incomplete or incorrectly handled. A total of 20 individuals completed the study with full PK data and were included in the analysis. Participants’ baseline characteristics are illustrated in Table 1. After enrolment, patients received an average amikacin dose of 10.9 (Range 9.4 -18.9) mg/kg bodyweight. The individual concentration-time curves, predicted using the three serum concentrations and the 1-compartment model for amikacin provided in the software MW/Pharm, are shown in Fig. 1. Summary PK parameters derived from the model are shown in Table 2.

**Table 2:** Pharmacokinetic parameters (n = 20).

Parameters	Median (IQR)
Clearance (L/h)	4.79 (4.03 - 5.75)
Volume of distribution (L)	16.30 (14.07 - 21.49)
AUC_0-24h_ (h x mg/L)	125.15 (106.73 - 174.46)
Cmax (mg/L)	27.8 (22.9 - 48.7)

Interquartile range (IQR), median observed 24-h area under the concentration–time curve (AUC_0-24_), peak serum concentration (Cmax).

Multivariable linear regression analyses were performed to explore the influence of demographic and clinical covariates on pharmacokinetic parameters (Appendix 2). None of the baseline parameters showed a significant correlation with PK parameters. For clearance, BMI showed a trend toward a negative association (β-coefficient = − 0.61, 95% CI −1.27 - 0.05, p = 0.066). No relevant associations were observed between covariates and Vd, AUC, or Cmax. The median eGFR at baseline was 123.3 ml/min/1,73 m^2^ (Range 106.3 - 146.2) and 116 ml/min/1,73 m^2^ (Range 75.1 - 131.4) at follow-up on day 30, with a small but statistically significant decrease of 11.3 ml/min/1,73 m^2^ (CI 7.44 – 16.43 ml/min/1,73 m^2^, p = 0.00003). Logistic regression models showed no significant influence of amikacin dose on eGFR change (p = 0.95). One patient developed high-grade hearing loss after 30 days of daily administration of amikacin 11.3 mg/kg bodyweight. Another patient was transitioned to an oral regimen before completing the 30-day study period due to the early onset of second-degree hearing loss and was subsequently excluded from the analysis.

## 4 Discussion

To our knowledge, this is the first study to apply a limited sampling strategy based on a one-compartment model for the estimation of amikacin PK in a sub-Saharan African (SSA) population. Data on aminoglycoside pharmacokinetics in MDR/RR-TB patients from SSA are scarce. This stands in stark contrast to the high disease burden and the continued need for aminoglycosides in selected treatment regimens. This study therefore addresses an important knowledge gap by providing pharmacokinetic data from a population that is underrepresented in PK research. We aimed to evaluate whether the PK profile of amikacin in an Ugandan patient population is comparable to that of populations in the Global North. This is particularly relevant because TB patient populations in the Global South, especially in SSA, differ from those in Europe or North America with respect to body composition, genetic background, comorbidities, and treatment context. Understanding whether PK parameters differ meaningfully between settings is important for interpreting and applying existing dosing recommendations and exposure targets [1].

Amikacin has been extensively studied, with numerous publications addressing PK and therapeutic drug monitoring in various patient populations. Accordingly, estimates of target PK-values for clearance and volume of distribution from population-PK models vary substantially, with clearance ranging from 0.77 to 5.5 L/h and volume of distribution from 10.7 to 41.5 L^27^. Sampling strategies for dose adjustments differ: Some studies have employed traditional monitoring schemes using peak and trough levels [27]^,^ while others have developed more sophisticated schemes [22,23]. However, aminoglycoside treatment for months or even years is not used in indications other than MDR/RR-TB.

To our knowledge, there are essentially two studies that investigated TB-patients with amikacin treatment, one conducted in Botswana [16] and one in the Netherlands [22]. In these studies, PK parameters were calculated using either two samples drawn at 0.5 and 23.5 h post-administration [16] or at least three unspecified samples between doses [22]. While in the first study, a two-compartment model was fitted to the data, the investigators of the second study applied a one-compartment model, citing insufficient late-phase samples. Usually, one-compartment models best fit data which do not entail a late or trough sample [28], as it was the case in the present study. Accordingly, the therapeutic drug monitoring evaluation software MW/Pharm suggests a one-compartment model for routine evaluation of amikacin serum concentrations [29,30]. While in the study from Botswana [16], weight-adjusted doses between 15 and 25 mg/kg body weight were applied, in the study from the Netherlands [22] patients received a 400 mg fixed dose, translating to body-weight adjusted doses of only 5.68 - 7.02 mg/kg. In our study, doses of 10 -15 mg/kg were given. The model-derived amikacin clearance value in the study from Botswana (population mean 1.47 L/h, SD 0.58) differed from the estimates in the study conducted in the Netherlands (population mean 4.62 L/h (IQR 4.05–5.35)) and our study (4.79 L/h (IQR 4.03 - 5.75)). Since model selection has an influence on parameter estimates, this may be an explanation. Consequently, the volume of distribution estimates in the study from Botswana were lower for the central compartment (2.10 L) and both compartments together, compared to the study conducted in the Netherlands (12.0 (IQR 9.14–15.3)) and our study (16.30 (IQR 14.07 - 21.49)).

Our model-derived PK results and the results from the Netherlands showed overlapping interquartile ranges indicating that no clear-cut difference was present. All values for clearance and volume of distribution were within the ranges of values for the respective parameters in a review of different population PK studies [28]. Since AUC values are dependent on the administered dose and are calculated as dose/Cl in compartmental modeling, direct comparisons are not feasible. The higher AUC values observed in this study likely reflect the higher doses administered in comparison to the Dutch study. In contrast, the Cmax values observed in the present study were slightly higher and showed a higher variability than in the Dutch study [22]. This discrepancy is likely due to higher doses and lower body weight in our patient population. Although the mean age in the present study was higher (37 years vs. 26 years) and relative doses of amikacin were higher (10.9 mg/kg vs. 6.67 mg/kg), our findings essentially confirm the one-compartment model data from the Dutch study, while a two-compartment model [16] gave different results, as expected when different models are used.

The present study demonstrates that pharmacokinetic sampling and model-based parameter estimation using a limited sampling strategy are feasible in a healthcare setting with limited resources. However, generating such data in resource-constrained environments is challenging. The present work provides practical information on sampling strategies and modeling approaches that may be useful for future pharmacokinetic research in similar contexts.

The study has limitations. Even in the controlled setting of a clinical study, about one third of enrolled patients dropped out, mainly due to uncertainties concerning sample collection and handling. This highlights the challenges of conducting PK studies in this demanding setting and helps explain why such studies have rarely been performed previously. Recruitment was also slow due to the introduction of new drugs and frequent updates to international and national MDR-TB treatment guidelines during the enrolment period.

## 5 Conclusion

We studied pharmacokinetic data for amikacin in an Ugandan MDR-TB patient population using a limited sampling strategy and one-compartment modeling. Of particular interest were the estimated AUC_0-24h_ values, which have been suggested as a key parameter for assessing aminoglycoside exposure and toxicity risk. Data for AUC_0-24h_, as well as clearance, Vd, and Cmax, were comparable to those reported in Western Europe, suggesting that major differences in amikacin PK between this cohort and previously studied populations are unlikely. The study addresses an important gap in PK data from sub-Saharan Africa and demonstrates the feasibility of conducting model-based PK analyses using limited sampling in this setting. These findings contribute locally derived data that may inform future PK and therapeutic drug monitoring research in MDR-TB populations in resource limited settings.

Further studies with larger cohorts and clinical outcome data are needed to evaluate the relationship between amikacin exposure and toxicity and to determine the role of such sampling strategies in routine care dealing with challenging situations. This is particularly relevant in the context of emerging resistance to bedaquiline and other new compounds, where aminoglycosides may again become indispensable as last-resort agents in selected patients. In such settings, careful and evidence-based use of aminoglycosides based on PK-data will be essential for minimizing debilitating drug toxicities in regions with the highest MDR/XDR-TB burden.

## CRediT authorship contribution statement

**Jan Hongler:** Writing – original draft, Investigation, Formal analysis, Data curation. **Sabine Haller:** Writing – review & editing, Supervision, Resources, Project administration, Methodology, Investigation, Funding acquisition, Data curation, Conceptualization. **Akello Susan Adakun:** Investigation. **Nina Lutz:** Supervision, Project administration, Methodology, Investigation. **Allan Buzibye:** Investigation, Data curation. **Marisa Kälin:** Writing – review & editing, Methodology, Investigation, Conceptualization. **Barbara Castelnuovo:** Writing – review & editing. **Christine Sekaggya-Wiltshire:** Writing – review & editing, Investigation. **George Abongomera:** Methodology, Investigation. **Alexander Jetter:** Writing – review & editing, Visualization, Supervision, Investigation, Formal analysis, Conceptualization. **Jan Fehr:** Writing – review & editing, Supervision, Project administration, Methodology, Investigation, Funding acquisition, Conceptualization.

## Declaration of competing interest

The authors declare that they have no known competing financial interests or personal relationships that could have appeared to influence the work reported in this paper.

## Figures and Tables

**Table:** 

Parameters	Covariate	β – Coefficients (95% CI)	p-value
Clearance (L/h)	Male sex (vs female)	−0.66 (−2.97, 1.66)	0.551
	Body weight (kg)	0.18 (−0.04, 0.40)	0.106
	BMI (kg/m^2^)	−0.61 (−1.27, 0.05)	0.066
	HIV positive (vs negative)	−0.22 (−2.21, 1.76)	0.813
	eGFR	0.02 (−0.06, 0.10)	0.573
Vd (L)	Male sex (vs female)	4.40 (−5.74, 14.53)	0.366
	Body weight (kg)	0.11 (−0.87, 1.09)	0.809
	BMI (kg/m^2^)	−0.27 (−3.15, 2.62)	0.845
	HIV positive (vs negative)	1.45 (−7.24, 10.14)	0.724
	eGFR	0.18 (−0.15, 0.52)	0.261
AUC_0-24h_ (h x mg/L)	Male sex (vs female)	0.53 (−121.65, 122.72)	0.993
	Body weight (kg)	−2.07 (−13.86, 9.73)	0.711
	BMI (kg/m^2^)	18.34 (−16.44, 53.13)	0.275
	HIV positive (vs negative)	48.20 (−56.54, 152.94)	0.338
	eGFR	0.48 (−3.55, 4.52)	0.800
Cmax (mg/L)	Male sex (vs female)	−8.33 (−37.60, 20.95)	0.550
	Body weight (kg)	0.19 (−2.64, 3.01)	0.888
	BMI (kg/m^2^)	2.22 (−6.12, 10.55)	0.576
	HIV positive (vs negative)	3.01 (−22.08, 28.11)	0.800
	eGFR	−0.06 (−1.03, 0.91)	0.896

Volume of Distribution (Vd), Confidence Interval (CI), median observed 24-h area under the concentration–time curve (AUC_0-24_), peak serum concentration (Cmax).

## Appendix 1 Mass spectrometry methodology

For the serum measurement of amikacin an in-house liquid chromatography mass spectrometry was used. Amikacin and the internal standard (gentamicin) were extracted from plasma samples using protein precipitation with acetonitrile. Chromatography was performed on Accucore HILIC 50 mm × 2.1 mm column. Mobile phase A consisted of 0.5% formic acid in acetonitrile, and mobile phase B consisted of 0.5% formic acid in water. Initial conditions of the mobile phase consisted of 85% mobile phase A for 1 min, reducing in organic content to 20% mobile phase A in 4.0 min, and equilibrated back to the initial conditions over a total run time of 7.0 min. The flow rate was 0.7 mL/min. The 3-D ion trap mass spectrometer (LCQ-FLEET; Thermo Scientific, Waltham Massachusetts, USA) was operated in positive ionization mode, and detection and quantification was performed using selective reaction monitoring (SRM) using transitions 425 and 322 for amikacin and the internal standard respectively. The assay was validated over a calibration range of 0.5 to 100 mg/L. The method was validated according to the published FDA guidelines for bioanalytical method development and validation within batch precision (%CV) of quality control samples was 6.42 to 10.05%, and the batch-to-batch precision was 10.89 to 11.80%. Accuracy was 95-102%. The samples were stable for at least 6 h on the bench and 48hrs in the auto sampler.
